# Attention mechanism enhanced LSTM with residual architecture and its application for protein-protein interaction residue pairs prediction

**DOI:** 10.1186/s12859-019-3199-1

**Published:** 2019-11-27

**Authors:** Jiale Liu, Xinqi Gong

**Affiliations:** 1 Mathematics Intelligence Application Lab, Institute for Mathematical Sciences, Renmin University of China, No. 59 Zhongguancun Street,Haidian District, Beijing, China; 2 Center for Mathematical Sciences and Applications,Harvard University, Boston, MA02138 USA

**Keywords:** Residual architecture, Attention, LSTM, Protein-protein interaction prediction, Monte Carlo

## Abstract

**Background:**

Recurrent neural network(RNN) is a good way to process sequential data, but the capability of RNN to compute long sequence data is inefficient. As a variant of RNN, long short term memory(LSTM) solved the problem in some extent. Here we improved LSTM for big data application in protein-protein interaction interface residue pairs prediction based on the following two reasons. On the one hand, there are some deficiencies in LSTM, such as shallow layers, gradient explosion or vanishing, etc. With a dramatic data increasing, the imbalance between algorithm innovation and big data processing has been more serious and urgent. On the other hand, protein-protein interaction interface residue pairs prediction is an important problem in biology, but the low prediction accuracy compels us to propose new computational methods.

**Results:**

In order to surmount aforementioned problems of LSTM, we adopt the residual architecture and add attention mechanism to LSTM. In detail, we redefine the block, and add a connection from front to back in every two layers and attention mechanism to strengthen the capability of mining information. Then we use it to predict protein-protein interaction interface residue pairs, and acquire a quite good accuracy over 72%. What’s more, we compare our method with random experiments, PPiPP, standard LSTM, and some other machine learning methods. Our method shows better performance than the methods mentioned above.

**Conclusion:**

We present an attention mechanism enhanced LSTM with residual architecture, and make deeper network without gradient vanishing or explosion to a certain extent. Then we apply it to a significant problem– protein-protein interaction interface residue pairs prediction and obtain a better accuracy than other methods. Our method provides a new approach for protein-protein interaction computation, which will be helpful for related biomedical researches.

## Background

Recurrent neural network(RNN), proposed by Hochreiter, is a major neural network in deep learning, which does as a bridge to connect the the information from past to present. It is based on the back propagation algorithm and contains the factor caused by time, therefore RNN is a kind of back propagation through time(BPTT) algorithm. What’s more, it can tackle the sequencial data including temporal and spatial data owing to its property.

**Fig. 1:**
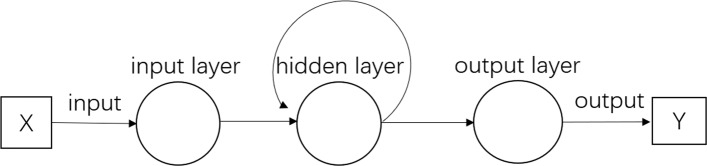
There is a standard RNN model, including three layers-input, recurrent, and output layer, whose outputs will be activated by linear or nonlinear functions acting on previous or latter inputs. The arrows show the flow in detail

Look at the standard RNN Fig. 1, the information is forward propagation from inputs to outputs. We can describe those information flow by a series of equations. Symbols and notations in this paper mainly refer to the book [1] written by Alex Graves. But here we’ll write it briefly. *x* denotes the input vector value, $x_{i}^{t}$ denotes the value of input *i*^*th*^ of vector *x* at time t, and *w*_*ij*_ denotes the weight from the unit *i* to unit *j*. For the hidden layer unit h, we denote the input of hidden layer unit h at time t:

1 $$ a_{h}^{t} = \sum\limits_{i=1}^{I}w_{ih}x_{i}^{t}+ \sum\limits_{h'=1}^{H}w_{h'h}b_{h'}^{t-1},  $$

the output of the hidden layer unit *h* at time t is denoted as $b_{h}^{t}$, and the activation function is *θ*_*h*_, so

2 $$ b_{h}^{t}=\theta(a_{h}),  $$

the output layer’s input can be calculated at the same time:

3 $$ a_{k}^{t} = \sum\limits_{h=1}^{H}w_{hk}b_{h}^{t}.  $$

Like the standard back propagation algorithm, BPTT is also a repeated application of chain rule. For the gradients of loss functions in RNN, the influence from loss function to hidden is not only through hidden layer’s output, but also through its next time step:

4 $$ \delta_{h}^{t} = \theta'(a_{h}^{t})\left(\sum\limits_{k=1}^{K}\delta_{k}^{t}w_{hk}+\sum\limits_{h'=1}^{t+1}w_{hh'}\right),  $$

where

5 $$ \delta_{j}^{t}\stackrel{def}{=}\frac{\partial \mathcal{L}}{\partial a_{j}^{t}},  $$

Then we can get the derivative of whole network weight respectively :

6 $$ \frac{\partial\mathcal L}{\partial w_{ij}}=\sum\limits_{t=1}^{T} \frac{\partial\mathcal L}{\partial a_{j}^{t}}\frac{\partial a_{j}^{t}}{\partial w_{ij}}=\sum\limits_{t=1}^{T}\delta_{j}^{t} b_{i}^{t}.  $$

**Fig. 2:**
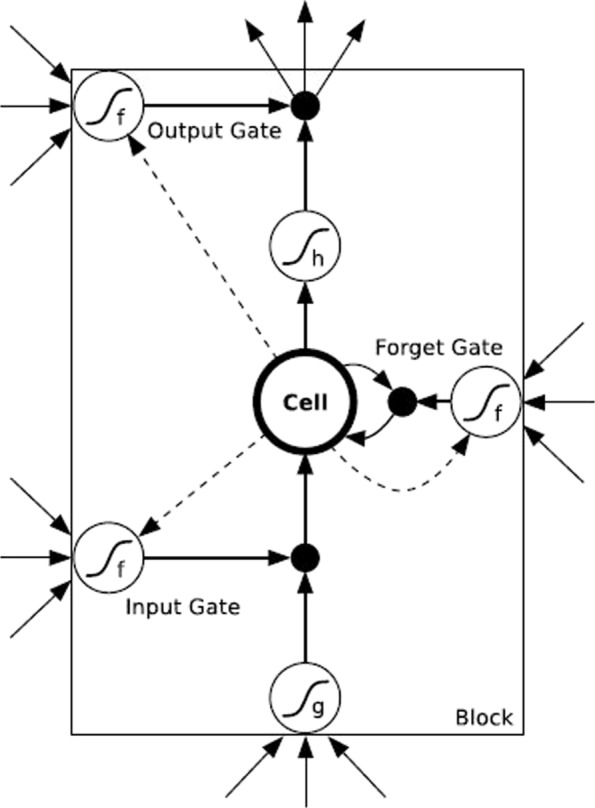
The memory block with one cell of LSTM neural network

Long short term memory [2](LSTM), as a variant of RNN, proposed by Hochreiter and shown in Fig. 2, consists of one block which has three gates(input/forget/output gate) whose every activation probability is from 0(the gate closes)to 1(the gate opens), and some cells which can remember information and transit it to the next step, while the hidden layer unit in RNN is replaced by three gates. The output values of input gate and forget gate are determined by the prior cells states and the input values.

The subscripts *ι*,*ϕ* and *ω* denote the input, forget and output gate of the block respectively, and *c* denotes one of the *C* memory cells. The peephole weight from cell *c* to the input, forget and output gates is denoted as *w*_*cι*_,*w*_*cϕ*_ and *w*_*cω*_ respectively. $s_{c}^{t}$ denotes the state of cell c at time t. *f*, *g* and *h* is the activation function of the gates, cell input and output, respectively. Let I denote the number of inputs, K denote the number of outputs and H denote the number of cells in the hidden layer.

Viewing to the Fig. 2 framework, we can get the equations :

input gate

7 $$ a_{\iota}^{t} =\sum\limits_{i=1}^{I}w_{i\iota}x_{i}^{t} + \sum\limits_{h=1}^{H}w_{h\iota}b_{h}^{t-1} + \sum\limits_{c=1}^{C}w_{c\iota}s_{c}^{t-1},  $$

8 $$ b_{\iota}^{t}=f\left(a_{\iota}^{t}\right),  $$

forget gate

9 $$ a_{\phi}^{t} =\sum\limits_{i=1}^{I}w_{i\phi}x_{i}^{t} + \sum\limits_{h=1}^{H}w_{h\phi}b_{h}^{t-1} + \sum\limits_{c=1}^{C}w_{c\phi}s_{c}^{t-1},  $$

10 $$ b_{\phi}^{t}=f(a_{\phi}^{t}),  $$

cell

11 $$ a_{c}^{t}=\sum\limits_{i=1}^{I}w_{ic}x_{i}^{t} + \sum\limits_{h=1}^{H}w_{hc}b_{h}^{t-1},  $$

12 $$ s_{c}^{t}=b_{\phi}^{t} s_{c}^{t-1}+b_{\iota}^{t}g\left(a_{c}^{t}\right),  $$

output gate

13 $$ a_{\omega}^{t} =\sum\limits_{i=1}^{I}w_{i\omega}x_{i}^{t} + \sum\limits_{h=1}^{H}w_{h\omega}b_{h}^{t-1} + \sum\limits_{c=1}^{C}w_{c\omega}s_{c}^{t-1},  $$

14 $$ b_{\omega}^{t} =f\left(a_{\omega}^{t}\right),  $$

cell’s output

15 $$ b_{c}^{t}=b_{\omega}^{t} h\left(s_{c}^{t}\right).  $$

**Fig. 3:**
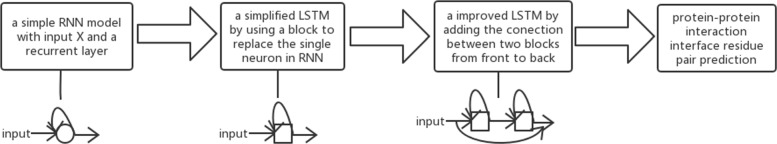
The evolutional flow processes from methods to application in this paper

When compared with RNN, LSTM is easier to change the weight of self-recursive model dynamically by adding the gates, and handle different scale data with better performance. Although there are many variants of LSTM, like GRU [3] which is a simplification of LSTM, and bidirectional LSTM [4], showing stronger performance, there are also some problems in LSTM–gradient explosion or gradient vanishing. [5, 6] both mentioned that in their paper, and employed residual learning [7] to avoid that problem, and did related experiment in speech and human activity recognition. That is why the applications of LSTM that we see are always in shallow neural networks. Though there are a lot of methods [8, 9] getting away from gradient explosion or gradient vanishing to some extent, such as weight regularization, batchnorm, clip gradient, etc, there are no better measures to solve the problem of gradient combining with layer scales. Recently, Sabeek [10] had done RNN in the depths of residual learning, which solved the gradient vanishing problem and showed a better performance. Given the thought of convolutional residual memory networks [11] and deep residual neural networks [7], we utilize a method with mathematical derivation to avoid the problems and deepen LSTM neural networks to excavate more information from original data in next section. Though some researchers aforementioned utilized this thought, there are some differences from our work–we use every two layers as a residue instead of one layer as a residue to accelerate the computational velocity in a sequential and larger dataset while Sabeek used it for sentimental analysis with a small dataset. And we prove its convergence theoretically. Furthermore, we utilize the attention mechanism to strengthen the extraction of information. This part will be shown in “Model architecture” section. If there are some notations you feel confused in “Results” section, we suggest that you’d better to read the “Methods” section before “Results” section. All of these will be described in the flow processes of the algorithm and application in our paper in Fig. 3.

## Results

Because the impact to accuracy of FRPP of layer number in neural networks is usually more uncomplicated and efficient than units numbers in parametric numbers. Like the methods of dichotomization, we use different layer numbers in a wide bound to find one with the best performance, then in this way continue to find the neighbor layer numbers and choose the optimal unit number. Viewing to the Table 1 left, we find that layer_60, not only the predicted true positive amounts in top 1%0 but also the mean accuracy, shows better performance than others. In like manner the unit _*n* and the model layer_*m*_unit_*n* can be denoted similarly in whole passage. After that, we continue to narrow it. Table 1 right shows the layer number near to layer_60, which is better than ones around it. So we next search the optimal unit number in layer_60, and finally we choose the best result with unit number in layer_60. Based on Table 1, Table 2 shows the results of the number of different units in detail. Despite the model mean of layer _60_*unit*_6 is lower than layer _60_*unit*_8, the number of RFPP(1%0) is quite lager inversely. Table 3 elaborates the result of model layer _60_*unit*_8 further on. In this model we can predict 8/11 if we choose the top 1%0 pairs of every dimer in the test set as predictions.

**Table 1:** The accuracy order of dimers in test set

Accuracy order	layer _10	layer _20	layer _30	layer _40	layer _50	**layer _60**	layer _70	layer _56	layer _58	layer _59	layer _60	layer _61	layer _62
1H9D	0.002534	0.003481	0.000013	0.000040	0.000067	**0.000053**	0.000747	0.003801	0.001147	0.000854	0.000053	0.017938	0.001227
1GL1	0.018904	0.006083	0.012480	0.000708	0.003592	**0.005086**	0.008416	0.011222	0.000105	0.001363	0.005086	0.005034	0.000026
2G77	0.009398	0.006355	0.002103	0.000076	0.001325	**0.000636**	0.000098	0.002614	0.001325	0.000443	0.000636	0.000210	0.002914
2VDB	0.000991	0.000991	0.002419	0.000091	0.001487	**0.000202**	0.000417	0.002680	0.001213	0.000972	0.000202	0.000913	0.004108
1KTZ	0.011788	0.006598	0.004096	0.007914	0.014994	**0.002094**	0.022055	0.060532	0.005134	0.003077	0.002094	0.034992	0.004874
1S1Q	0.003033	0.002597	0.000437	0.002757	0.000827	**0.000758**	0.001126	70.001815	0.003699	0.006112	0.000758	0.000184	0.009720
1BUH	0.000137	0.002547	0.001425	0.010694	0.007806	**0.009923**	0.000742	0.004908	0.003434	0.001229	0.009923	0.016499	0.000185
1BKD	0.003846	0.000317	0.002938	0.002416	0.000311	**0.000227**	0.000386	0.000053	0.000945	0.002301	0.000227	0.000724	0.001468
1GPW	0.000556	0.000281	0.004957	0.001203	0.001449	**0.000386**	0.000311	0.002241	0.000160	0.000226	0.000386	0.000647	0.000496
1SYX	0.000989	0.006525	0.000537	0.000141	0.001271	**0.000876**	0.001864	0.001328	0.000141	0.009181	0.000876	0.001977	0.002740
1Z5Y	0.029783	0.001220	0.001341	0.000157	0.006787	**0.000254**	0.003635	0.001981	0.004903	0.008816	0.000254	0.000157	0.002778
mean	0.007451	0.003363	0.002977	0.002382	0.003629	**0.001863**	0.003618	0.008470	0.002019	0.003143	0.001863	0.007207	0.002776

Note: mean means the average of columns and the bold fonts are the minimal mean values of the corresponding model and the layer _*m* means that the layer number is m

**Table 2:** The accuracy order of dimers in test set with layer _60

Accuracy order	unit _5	unit _6	unit _7	unit _8	unit _9
1H9D	0.002574	0.000293	0.000373	0.000053	0.006642
1GL1	0.006397	0.000419	0.000052	0.005086	0.000629
2G77	0.000336	0.004471	0.003813	0.000636	0.006704
2VDB	0.000848	0.000339	0.008646	0.000202	0.000711
1KTZ	0.014790	0.001890	0.015494	0.002094	0.004689
1S1Q	0.024311	0.001287	0.006916	0.000758	0.001677
1BUH	0.000751	0.000332	0.000703	0.009923	0.003493
1BKD	0.003591	0.001284	0.007017	0.000227	0.000078
1GPW	0.002180	0.000311	0.000401	0.000386	0.000571
1SYX	0.005085	0.004633	0.035678	0.000876	0.001215
1Z5Y	0.004928	0.001135	0.000556	0.000254	0.007379
mean	0.005981	0.001490	0.007241	0.001863	0.003072

**Table 3:** The prediction results of layer _60_*unit*_8 in test set

PDB Code	1H9D	1GL1	2G77	2VDB	1KTZ	1S1Q	1BUH	1BKD	1GPW	1SYX	1Z5Y
Protein function	OX	EI	OG	OX	OR	OX	EI	OG	OX	OX	ES
RFPP	4	194	142	31	113	33	1017	73	77	31	21
Number of surface residue pair	74980	38141	223440	153360	53955	43520	102490	321630	199500	35400	82800
Accuracy order(%0)	0.053	5.086	0.636	0.202	2.094	0.758	9.923	0.227	0.386	0.876	0.254
NCPD	1%0			3 *%%*				8 *%%*			
	8			4				7			
Number of interface residue pair	501	300	425	382	188	245	301	687	434	210	264
Random experiment	141	124	442	364	274	173	317	413	401	165	296

Note: NCPD(m%0)=n means that there are n dimers which meet the in equation accuracy order ≤ m%0, and the result of last row will be explained in next section

### Comparison with other methods

PPiPP [12] is a method by using protein sequences for monomer binding site predictions, and PAIRpred [13] is a fresh complex interface prediction approach published in 2014 and realizes a higher prediction accuracy. Zhenni Zhao [14] used a deep learning architecture–multi-layer LSTMs, to predict interface residue pairs, and achieved a better accuracy. Table 4 shows the results from the above-mentioned approaches in different Docking Benchmark Data dataset. The evaluation index is RFPP. When p equals 90%, our model can predict around 90*%* proteins correctly in our dataset if we choose top 194 residue pairs as prediction. And it improves around a third when comparing with others. Because of the differences of proteins that we select in our train and test set, and pre-treatment methods, we can only take a look at the results of the comparison partly. In addition, our protein sequence is longer and residue pairs amount is bigger than above, hence these can increase the difficulties for predicting RFPP. In order to balance the comparison, we use another evaluation index–accuracy order, to replace it. Wei Wang.etc [15] used different machine learning methods chosen by different protein properties to predict interface residue pairs. we show the comparison and our prediction precision by choosing top 1%0 residue pairs in Table 5.

**Table 4:** Comparison with PAIRpred, PPiPP and multi-layered LSTM

Data set	Method		RFTP(p)				
			10%	25%	50%	75%	90%
DBD 3.0	PPiPP		9	19	78	297	760
	PAIRPred						
	PAIRPred _1	No post-processing	2	13	68	257	804
	PAIRPred _2	No post-processing	1	5	22	89	282
		With post-processing	1	3	16	103	272
DBD 4.0	PAIRPred _2	No post-processing	2	6	19	75	340
		With post-processing	1	3	18	101	282
DBD 5.0	Multi-layered LSTM Network	lstm _1_*nodes*_20	12	53	139	175	331
		lstm _5_*nodes*_20	13	17	46	146	271
		lstm _6_*nodes*_35	1	2	7	639	1384
		lstm _5_*nodes*_45	4	13	36	94	847
	our model	layer _60_*unit*_8	4	31	33	113	194

Note: lstm _*m*_*nodes*_n means the model has m layer LSTMs,and each layer has n units

**Table 5:** Comparison by choosing top 1%0 residue pairs

Methods	Precision
multi-layer LSTM[14]	30.8%
different machine learning[15]	42.4%
our model	72.7%

Furthermore, we also use random theory to calculate the RFPP. As we know mathematical expectation is one of the most significant numerical characteristics to describe the average of variables. *X* denotes the random variable of RFPP here. In order to correspond to our index of algorithm, we select 1000 pairs randomly, so

$$P(X=i)= \left \{ \begin{aligned} &\frac{C_{N-M}^{i-1}C_{M}^{1}C_{N-M-i}^{1000-i}}{C_{N}^{1000}}, \quad i=1,2,...,1000 \\ &1-\sum\limits_{i=1}^{1000} \frac{C_{N-M}^{i-1}C_{M}^{1}C_{N-M-i}^{1000-i}}{C_{N}^{1000}}. \quad else \end{aligned} \right.  $$

where N denotes the number of surface residue pairs and M denotes the number of interface residue pairs.

Then

$$ E(X)= \sum\limits_{i} i\times P(X=i)\ge \sum\limits_{i=1}^{1000} i\times P(X=i)+1000\times \frac{C_{N-M}^{1000}}{C_{N}^{1000}}  $$

Why we use the inequality is that the the latter is simpler than the former in computational complexity, but calculation is still complicated based on pure theory. Monte Carlo simulation is a well-known method to compute the expectation by using the frequency of events to estimate its probability respectively. This will be more convenient for us to achieve them. We use, more specifically, random simulation about 10 billion times, then we count it that happens respectively. The formula:

$${\begin{aligned} \sum\limits_{i=1}^{i=1000}i &\times\frac{\mathrm{count(RFPP=\mathit{i})}}{10 \text{billion} }+1000\\ &\times\frac{10 \text{billion}-\sum\limits_{i=1}^{1000}\mathrm{count(RFPP=\mathit{i})}}{10\text{billion}}=\frac{1}{10 \text{billion}}[\cdots] \end{aligned}} $$

Here,the purpose we extract the coefficient $\frac 1{10 \text {billion}}$ is to avoid something happening to reduce the error like the frequency $\frac {15}{10 \text {billion}}$ limited to 0. All the results will be shown in the last row of Table 3. We can clearly see that our result is extremely better than random RFPP except 1GL1 and 1BUH.

## Discussion

**Fig. 4:**
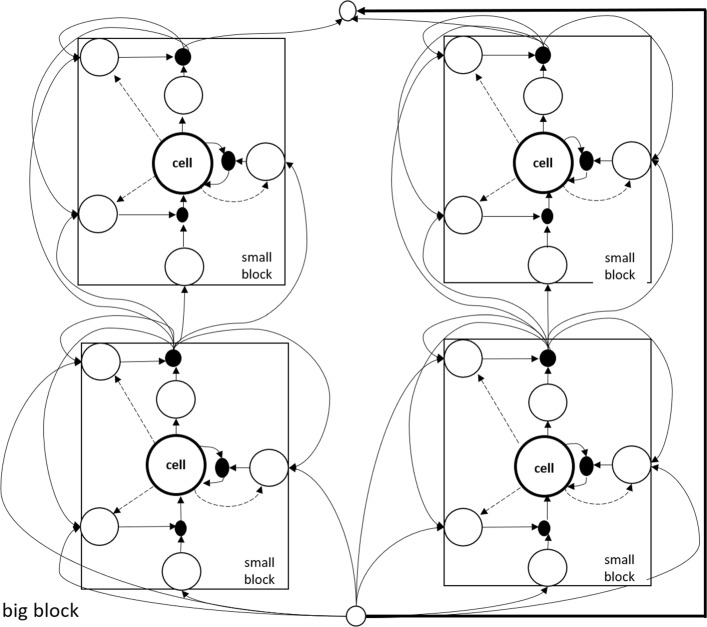
Prediction of different model parameters, where code _*m*_n means the layer number of LSTM is n, and the unit number in each LSTM layer is m. Longitudinal axis represents accuracy order and horizontal axis means PDB respectively

**Fig. 5:**
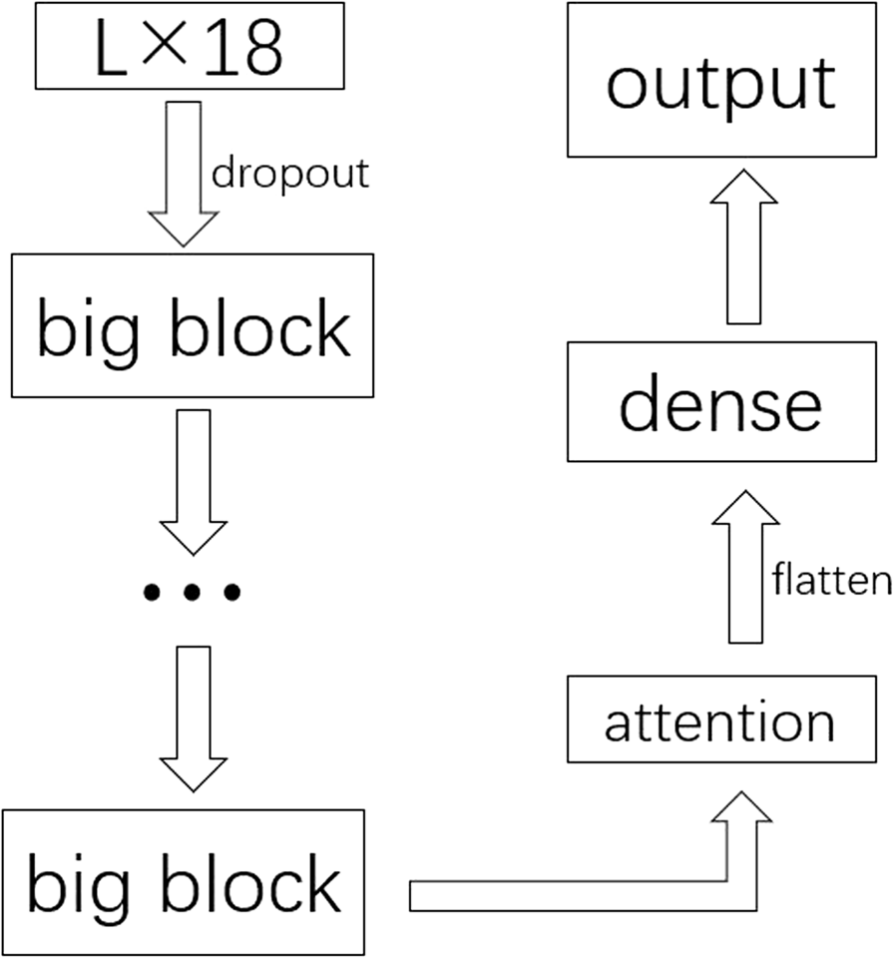
Model architecture. Where big block LSTM is defined as mentioned above

Viewing Tables 1 and 2, we select the two best prediction accuracy in each table while choosing top 1%0 as estimated index. According to the Fig. 4, we find that our model shows poor performance in protein 1BUH and good performance in protein both 2VDB and 1Z5Y commonly. One of the most possible reasons is that 1BUH is far away from the train data in homology while 2VDB and 1Z5Y aren’t. This will be verified by identity matrix to some extent which shows the highest homology in train set is 12.86% between 1DFG and 1BUH. As for 1GL1, We notice that the random model with RFPP 124 shows better performance than our model with RFPP 194. This is hard to give an explanation. But from the perspective of homology, we find that 1GL1 has a little higher homology 16.7% with 2I9B. This may be one possible reason for 1GL1. We also depict some of protein-protein interaction interface pairs predicted by our model in Fig. 5 where the first row is predicted well, but the second is not.

On the one hand, how to choose hyperparameters is also a complicated problem in deep learning. The existing methods such as grid search which gives a trick for us. On the other hand, most biological data will lose some information when we transform it. In detail we use three-dimensional coordinates of one atom to replace an amino acid for simplification and we excessively depend on the structure of monomers, It’s one of the biggest limitations. Because our problem is to predict whether any two monomers can form a dimer complex. And the different features selection from original data make different prediction performance. If we don’t consider any physicochemical and geometric properties, from sequence to predict structure directly usually shows low accuracy. And because our prediction method depends on the 9 feature values from monomers structure other than dimer complexes structure, therefore if some values are missing, we will delete the corresponding pairs or whole dimers. This is also a limitation. Recently AlQuraishi [16] employ bi-directional LSTM to predict protein structure from protein sequence and obtain state-of-art achievement. This may inspire us to rethink the problem from protein sequence perspective. Data extreme imbalance is a serious problem introduced to model for training. How to choose a good approach is also preferred.

## Conclusions

In this paper, we employ a novel LSTM based on residual architecture and attention mechanism, and derive the gradient. Then we utilize this model to predict protein-protein interaction interface residue pairs, and compare our model with standard LSTMs and other methods, to show that our prediction accuracy is more than 72 percent which far surpasses other methods in performance. This will be more significant for biomedical related research as well as the computational though there are a lot of further problems we can consider like the feature selections, coevolution [17] information, contact preferences and interface composition [18].

## Methods

### Algorithm derivation

**Fig. 6:**
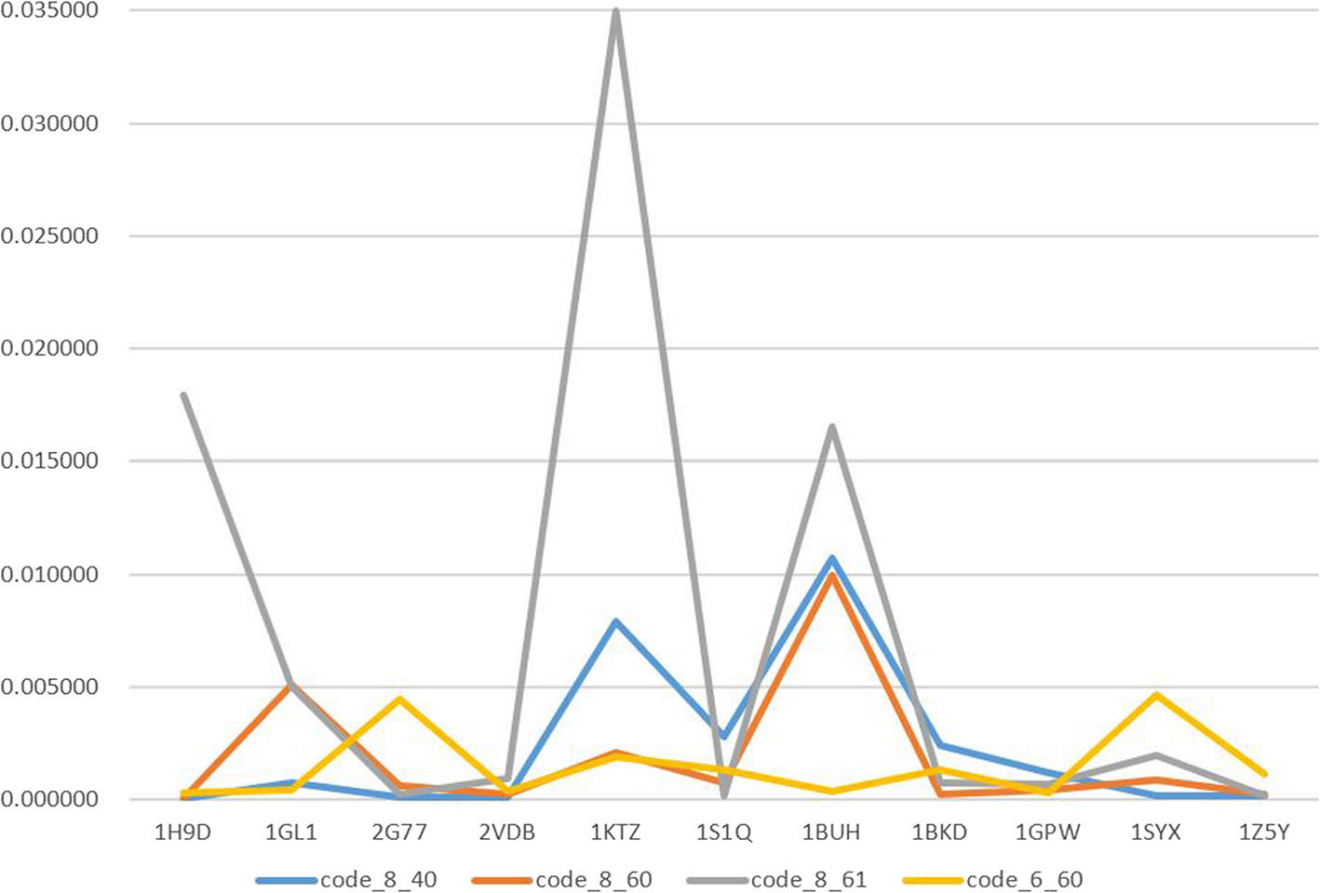
Some of prediction of protein-protein interaction interface residue pairs, which are highlighted in surface and shown in different colors with amino acid name and site in corresponding chains. **a** 1H9D **b** 2VDB **c** 1GL1 **d** 1BUH

Before deriving the equations of backward pass, we need to redefine LSTM. We call the LSTM unit a small block, and the two LSTM layers a big block, which possesses an additional connection from the output layer *l* to the output layer *l+2* (see bold line in Fig. 6).

Figure 6 is a simplified version, and we just consider that there is only one cell in LSTM unit. However, what we usually use is full connection traditionally. In order to view the differences from different layers, we use the (·)^*l*^ to present the values of the layer *l* respectively. For example, the $\left (b_{c}^{t}\right)^{\mathit {l}}$ denotes the cell output value of layer *l*. And if they are in a same layer, then we omit the superscript *l* additionally.

$$ \left(\epsilon_{c}^{t}\right)^{\mathit{l+2}}\stackrel{def}{=}\frac{\partial \mathcal L}{\partial \left(b_{c}^{t}\right)^{\mathit{l}}+\partial\left(b_{c}^{t}\right)^{\mathit{l+2}}}, \qquad \epsilon_{s}^{t}\stackrel{def}{=}\frac{\partial \mathcal L}{\partial s_{c}^{t}},   $$

cell’s output

16 $$ \epsilon_{c}^{t} = \sum\limits_{k=1}^{K}w_{ck}\delta_{k}^{t} + \sum\limits_{g=1}^{G}w_{cg}\delta_{g}^{t+1},  $$

output gate

17 $$ {\begin{aligned} \delta_{\omega}^{t}=\frac{\partial{\mathcal{L}}}{\partial a_{\omega}^{t}} &=\frac{\partial{\mathcal{L}}}{\partial\left(b_{c}^{t}\right)^{l}+\partial\left(b_{c}^{t}\right)^{l+2}} \frac{\partial \left(b_{c}^{t}\right)^{{l}}+\partial \left(b_{c}^{t}\right)^{} {l+2}}{\partial \left(b_{\omega}^{t}\right)^{l}}\frac{\partial\left(b_{\omega}^{t}\right)^{l}}{\partial\left(a_{\omega}^{t}\right)^{l}}\\ &= \left(\epsilon_{c}^{t}\right)^{l+2}h\left(s_{c}^{t}\right)\left(1+\frac{\partial\left(b_{c}^{t}\right)^{l+2}}{\partial\left(b_{c}^{t}\right)^{l}}\right)f'\left(a_{\omega}^{t}\right), \end{aligned}}  $$

state

18 $$ \epsilon_{s}^{t} = b_{\omega}^{t}h'\left(s_{c}^{t}\right)\epsilon_{c}^{t} + b_{\phi}^{t+1}\epsilon_{s}^{t+1}+ w_{c\iota}\delta_{\iota}^{t+1} + w_{c\phi}\delta_{\phi}^{t+1}+ w_{c\omega}\delta_{\omega}^{t},  $$

cell

19 $$ \delta_{c}^{t} =\frac{\partial \mathcal L}{\partial a_{c}^{t}}=\frac{\partial \mathcal L}{\partial s_{c}^{t}} \frac {\partial s_{c}^{t}}{\partial a_{c}^{t}}=\epsilon_{s}^{t}b_{\iota}^{t}g'\left(a_{c}^{t}\right),  $$

forget gate

20 $$ \delta_{\phi}^{t} = \frac{\partial\mathcal L}{\partial a_{\phi}^{t}}=\frac{\partial\mathcal L}{\partial s_{c}^{t}}\frac{\partial s_{c}^{t}}{\partial b_{\phi}^{t}}\frac{\partial b_{\phi}^{t}}{\partial a_{\phi}^{t}}=\epsilon_{s}^{t} s_{c}^{t-1}f'\left(a_{\phi}^{t}\right),  $$

input gate

21 $$ \delta_{\iota}^{t} =\frac{\partial\mathcal L}{\partial a_{\iota}^{t}}=\frac{\partial\mathcal L}{\partial s_{c}^{t}}\frac{\partial s_{c}^{t}}{\partial b_{\iota}^{t}}\frac{\partial b_{\iota}^{t}}{\partial a_{\iota}^{t}}= \epsilon_{s}^{t} g\left(a_{c}^{t}\right) f'\left(a_{\iota}^{t}\right).  $$

We can see that if gradient vanishing happens in layer *l*+2 which also means that $\frac {\partial \left (b_{c}^{t}\right)^{l+2}}{\partial \left (b_{c}^{t}\right)^{l}}=0$, the conventional LSTM fail to update parameters before layer *l*+2. But from (2.2), our model architecture can prohibit that because of $1+ \frac {\partial \left (b_{c}^{t}\right)^{l+2}}{\partial \left (b_{c}^{t}\right)^{l}}=1$.

### Background, data, and evaluation criteria

Proteins are the foundations of life activities for cells, but most of them exert their functions only having interaction with other molecules. As a result, protein-protein interaction prediction becomes a very important project. The first step of it is to know the site of interface residue pairs precisely. The most common methods are from experimental and computational perspective recently. One the one hand, anatomizing all proteins is unfeasible to experiment technicians for the high expenses. On the other hand, the computational methods become the scientific tidal current due to its low costs and convenience, such as template [19] and structure model [20] methods. In recent years, artificial intelligence especially machine learning and deep learning has been used in computer vision image and language recognition,etc, and received many achievements. At the same time some computational researchers transfer those methods to biology. Protein contact prediction [21] is one of the good instances by using deep residual networks. Though there are some achievements [13–15] in protein-protein interaction interface residue pairs predictions especially while Zhenni [14] used a deep learning architecture to tackle this project, we still need to proceed and develop new algorithms for its low accuracy. Here we will apply our method to predict interface residue pairs.

Our data is from benchmark versions 3.0, 4.0, and 5.0 [22, 23] on the international Critical Assessment of PRotein-protein Interaction predictions(CAPRI). All selected dimers whose states are unbound satisfy our requirement and add up to 54, then they are randomly split into three parts including train, validation, test set with ratio around 6:2:2 (shown in Table 6). Moreover, In order to illustrate test efficiency of our data partition structure, we identity multi protein sequences homology comparison in ClustalW2 https://www.ebi.ac.uk/Tools/msa/muscle/. Both of the results are attached in supplementary–identity matrix, and only the homology ≥30*%* of two dimers is shown in Table 6. From the identity matrix, we can see only the partition of 2I25(in train set) and 1H9D(in test set) is little unreasonable because of the homology with 40%, but we will show the better prediction result of 1H9D with such litter higher homology later. Every residue pair consists of 18 features which are concatenated by the two 9 feature values of each residue proposed basing on physicochemical and geometric properties which are common in computation. The 9 features are listed below and their computation are shown respectively in Table 7. Interior Contact area(IC) [24], Exterior Contact area with other residues(EC) [24] Exterior Void area(EV) [24, 25], Absolute Exterior Solvent Accessible area(AESA) [25], Relative Exterior Solvent Accessible area(RESA) [25], Hydropathy Index(HI, two versions) [26, 27] and pK _*α*_ (two versions) [28]. paper [29]summarized these features and their respective tools for computation. Here we just simply describe it. IC is the Interior Contact area between atoms inside a residue. EC is the Exterior Contact area between residues from the same protein. EV is the area does not contact with water molecules or any amino acid. AESA is the contact area between water molecules and surface residues.

**Table 6:** The data partition structure and homology (≥30*%*)

Train(32)	Validation(11)	Test(11)	Homology(%)
1UDI,1EWY,2SIC,2I25,7CEI,2I9B,1FFW,1ACB, 2J0T,1OC0,1Y64,2O3B,1MAH,1DFJ, 1R0R,1BVN, 2OUL,2ABZ,2A5T,2HLE,1GLA,1WQ1,1ATN,1GHQ, 2B42,1R6Q,1CLV,1KXQ,1IBR,1KAC, 1US7,1AK4	1OYV,2PCC,1CGI, 2AJF,1B6C,1MQ8, 1FC2,1AY7,1ZM4, 4CPA,1KXP	1H9D,1GL1,2G77, 2VDB,1KTZ,1S1Q, 1BUH,1BKD,1GPW,1SYX,1Z5Y	1KXQ,1BVN(tr,tr,98.59); 2I25,1H9D(tr,te,40); 2ABZ,4CPA(tr,va,97.72); 4CPA,1H9D(va,te,33.33); 2SIC,1OYV(tr,va,68.5); 1GPW,1H9D(te,te,33.33); 2SIC,1R0R(tr,tr,68.25); 1BUH,1H9D(te,te,33.33)

Note: A,B(C,D,E) in homology column means the homology between dimers A and B is E%, where C and D is the corresponding data partition structure of A and B.

**Table 7:** The 9 features and their computation

Features	Abbreviation	Software or Researchers
Interior Contact area	IC	Qcontacts
Exterior Contact area with other residues	EC	Qcontacts
Exterior Void area	EV	NACCES, Qcontacts
Absolute Exterior Solvent Accessible area	AESA	NACCES
Relative Exterior Solvent Accessible area	RESA	NACCES
Hydropathy index, version 1	H1	Jack Kyte et al.
Hydropathy index, version 2	H2	David Eisenberg
pKa1: computation	pKa1	PROPKA3.1
pKa2: standard	pKa2	PROPKA3.1

RESA is a proportion between AESA in protein and AESA of free amino acids. H1 and H2 are two versions of hydrophobicity index used to measure the hydrophobic ability. pKa is a reflection of the electrostatics of surface residue in the specific environment.

A residue pair is defined as interface if the contact areas of two amino acids from different two monomers are not zero. Here we use two statistical evaluation criteria combining biological meanings to measure our model prediction: rank of the first positive prediction(RFPP), and the number of correctly predicted dimers(NCPD). In order to overcome the length differences and balance the predicted difficult degree in different proteins, accuracy order is adopted.

$ accuracy \quad order = \frac {RFPP}{TNRP} $, where TNRP is the total number of residue pairs in a dimer.

### Model architecture

**Fig. 7:**
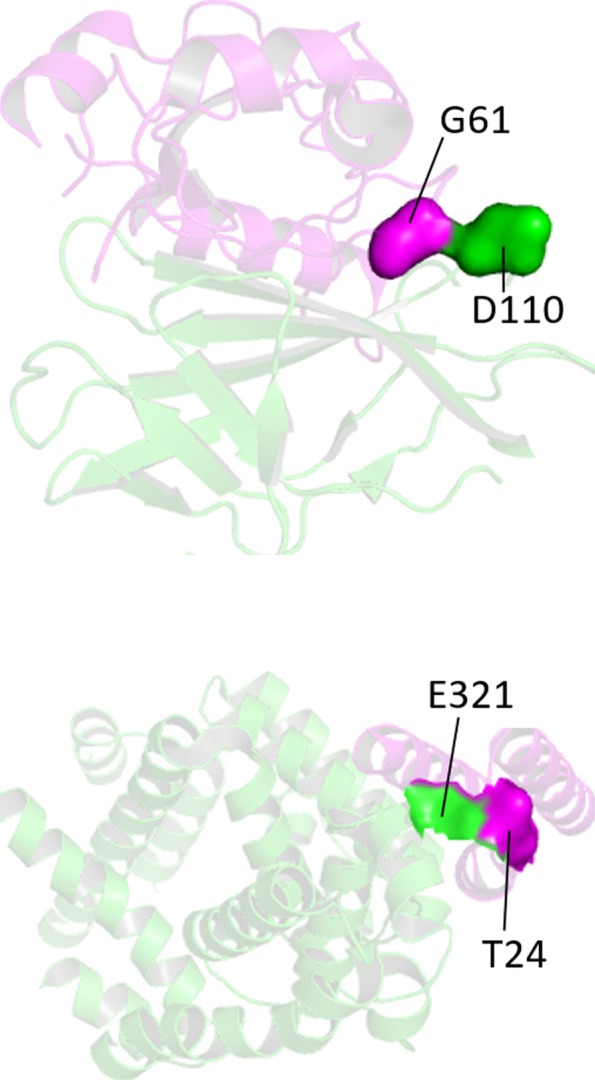
Big block LSTM with no connection from the same layers and full connection from adjacent two layer networks. To simplify the network, we just consider an input with one unit in the layer *l* and an output with one unit in the layer *l+2*

This is a binary classification problem. The input format is a matrix with dimension *L*×18 Fig. 7, since every amino acid consists of 9 features and a residue pair possesses 18 features. Where L is the number of combinations of amino acid residue pairs. We use the label 1 to present that the pair is an interface residue pair, and label 0 is opposite. Because the amount of label 0s is extremely larger than 1s, so we need to pre-treat the imbalance between the positive and negative samples. We use a distance to exclude some impossible residue pairs. The distance between different chains will be small to some way to meet a threshold if the residue pairs are contact. Therefore we choose the residue pairs with the most short distance, then choose 3 residues around them in each chain respectively, hence there are 3×3 pairs altogether. This method can reduce the amount of negative samples efficiently. Because we use this selective method which can make the data sequential, therefore the LSTM neural network is a quite good choice for us. Then the data pre-treated will be input to the neural network architecture. There are some hyperparameters to explain in detail. Dropout [30] is a way to prevent model from over-fitting, because it can be a probability from 0 to 1 to drop out the units and cutdown all the connections from the units to next units randomly. In this paper, we use 0.15 to dropout some redundant information of the inputs. According to the new achievement, Wojciech Zeremba [31] proposed a new method–adding dropout from the current layer to next layer, but not to recurrent layer, to regularize the RNN, which inspires us to use dropout in LSTM and fit it in 0.6. These hyperparameters can be fitted by a common technique–grid search, and the results will be shown in supplementary. Attention has been widely used in speech recognition [32] and reasoning [33],etc for its efficient mechanism which can reallocate weight and retrieve some more critical information, therefore these motivate us to use attention in our model. The dense layer’s activation function is softmax, and the loss function is categorical crossentropy. Softmax and crossentropy is designed as following

22 $$ \sigma(\mathbf{Z}_{j})=\frac{e^{z_{j}}}{\sum_{k=1}^{K}e^{z_{k}}} \quad for \,\, j=1,2,...,K.  $$

23 $$ H(p,q)=E_{p}[-\log q]=H(p)+D_{KL}(p\|q)  $$

where *p* is a true distribution while *q* is an estimated distribution. Softmax function can mapping a *n*^*d*^ vector to another *n*^*d*^ vector whose elements are from 0 to 1. Crossentrop, equal to maximum likelihood estimation, is an index to measure the gap between the true distribution and the estimated distribution.

## Data Availability

Our code and parameters of model can be found in https://github.com/Jiale-Liu/LSTM and data is available in ftp://202.112.126.135/pub/surrounding_3.mat.

## Abbreviations

BPTT: Back propagation through time
LSTM: Long short term memory
NCPD: The number of correctly predicted dimers
RFPP: Rank of the first positive prediction
RNN: Recurrent neural network
TNRP: Total number of residue pairs in a dimer
